# Light‐Induced Switching of Tunable Single‐Molecule Junctions

**DOI:** 10.1002/advs.201500017

**Published:** 2015-04-16

**Authors:** Torsten Sendler, Katharina Luka‐Guth, Matthias Wieser, Lokamani, Jannic Wolf, Manfred Helm, Sibylle Gemming, Jochen Kerbusch, Elke Scheer, Thomas Huhn, Artur Erbe

**Affiliations:** ^1^ Helmholtz‐Zentrum Dresden – RossendorfBautzner Landstraße 40001328DresdenGermany; ^2^ Department of PhysicsUniversität Konstanz78457KonstanzGermany; ^3^ Institute for Materials Science and Max Bergmann Center of Biomaterials DresdenTechnische Universität Dresden01062DresdenGermany; ^4^ Fachbereich ChemieUniversität Konstanz78457KonstanzGermany; ^5^ Center for Advancing Electronics DresdenTechnische Universität Dresden01062DresdenGermany; ^6^ Faculty of ScienceTechnische Universität Chemnitz09107ChemnitzGermany

**Keywords:** diarylethenes, in situ switching, molecular electronics, photochromism, single‐molecule junctions

## Abstract

A major goal of molecular electronics is the development and implementation of devices such as single‐molecular switches. Here, measurements are presented that show the controlled in situ switching of diarylethene molecules from their nonconductive to conductive state in contact to gold nanoelectrodes via controlled light irradiation. Both the conductance and the quantum yield for switching of these molecules are within a range making the molecules suitable for actual devices. The conductance of the molecular junctions in the opened and closed states is characterized and the molecular level *E*_0_, which dominates the current transport in the closed state, and its level broadening *Γ* are identified. The obtained results show a clear light‐induced ring forming isomerization of the single‐molecule junctions. Electron withdrawing side‐groups lead to a reduction of conductance, but do not influence the efficiency of the switching mechanism. Quantum chemical calculations of the light‐induced switching processes correlate these observations with the fundamentally different low‐lying electronic states of the opened and closed forms and their comparably small modification by electron‐withdrawing substituents. This full characterization of a molecular switch operated in a molecular junction is an important step toward the development of real molecular electronics devices.

## 1 Introduction

This is an open access article under the terms of the Creative Commons Attribution License, which permits use, distribution and reproduction in any medium, provided the original work is properly cited.

The field of molecular electronics aims at using the structural and electronic properties of single organic molecules for building electronic circuits on the nanometer scale with an unprecedented accuracy. Novel developments in nanostructuring and synthesis of suitable molecules during the past two decades have opened possibilities of reliably forming contacts to single molecular wires using proper anchoring groups contacting the molecules both mechanically and electrically to metallic electrodes.^1^, ^2^ The integration of electrically functional single molecules, which are structurally more complex than simple molecular wires, into metallic electrodes is still one of the major challenges of today's nanoelectronics. Moreover, the controlled manipulation of the electrical behavior of those nanosystems under the influence of external stimuli still needs to be proven, although such systems have seen great progress during the last few years. Besides the idea of affecting the electronic structure via electric^3^, ^4^, ^5^ or magnetic fields,^6^ a highly promising concept is switching via light irradiation.^7^, ^8^, ^9^, ^10^ Various photochromic molecules have been developed, including the large group of diarylethenes (DAE).^11^, ^12^

These molecules react on light irradiation by reversibly changing between two well‐defined states of different properties: a ring‐opened, nonconjugate “off”‐state and a ring‐closed, conjugate “on”‐state. Density‐functional‐based calculations and extensions by nonequilibrium Green's functions on short model structures indicate a switching ratio between the nonconducting off‐state and the conducting on‐state of approximately 100.^13^, ^14^, ^15^, ^16^ Multireference complete active space (CAS) augmented Hartree–Fock calculations on the central diaryl moiety have shown that light‐induced switching involves a complex sequence of electronic and structural relaxations, which exclude a simple thermal ring opening or closure.^11^, ^17^, ^18^, ^19^ Considering the DAE's thermal stability,^20^, ^21^ photoswitching characteristics and the variety of synthesizable modifications,^22^, ^23^, ^24^ they are highly interesting candidates for integrated molecular switches.

Previous work delivered already an accurate picture of the electrical behavior of diarylethene junctions assembled in the on‐state, as well as in the off‐state.^15^, ^25^, ^26^, ^27^ This comprises, for example, conductance values, strength of the electronic metal–molecule binding Γ, and molecular level *E*_0_. Due to difficulties of switching single DAE molecules in situ in direct contact to metallic nanoelectrodes, experimental findings regarding light‐induced switching have been mainly obtained from experiments on self‐assembled monolayers and ensembles of DAE studied on gold surfaces,^28^, ^29^ between gold nanoparticles,^30^ and single walled carbon nanotubes.^31^ Reversible switching of ensembles on surfaces or in a solution was successfully shown pointing out the high potential of DAE acting as electronic devices. Evidence of in situ switching of single DAE between gold contacts has been demonstrated in an irreversible ring‐opening process in a mechanically controllable break junction (MCBJ) setup,^9^ and statistical evidence for ring‐opening as well as ring‐closing has been gathered via scanning tunneling microscope studies.^32^ In these studies, changes in the electrical resistance were used to distinguish between the two states. A combination of optical and thermal excitations^17^ was used to achieve reproducible in situ switching between the two conduction states of dimethyldihydropyrene‐type molecules.^21^ Alternative mechanisms leading to a switching of the conductance, such as chemo‐electrical switching and an increase of conductance during irradiation with UV light, were demonstrated in spiropyran derivatives.^33^

When developing a concept for reliable switching of molecular switches, the influence of the metal electrodes on the molecular states and their switching behavior needs to be controlled.^34^ We have, therefore, developed a new design for switchable molecular wires (SMWs) to increase the transfer rate from the excited state of the inner ring during the switch closing to the final on‐state.^22^ Here, we show that the newly developed switch exhibits a large conductance value in the on‐state and that switching of molecules, which are in electrical contact with the electrodes, from the off‐state to the on‐state is possible. Remarkably, the reverse switching from the on‐state to the off‐state is suppressed in our structures because the transition cannot be performed when the SMWs are mechanically anchored to gold electrodes.

## 2 Results and Discussion

### 2.1 Switchable Molecular Wire

**Figure 1:**
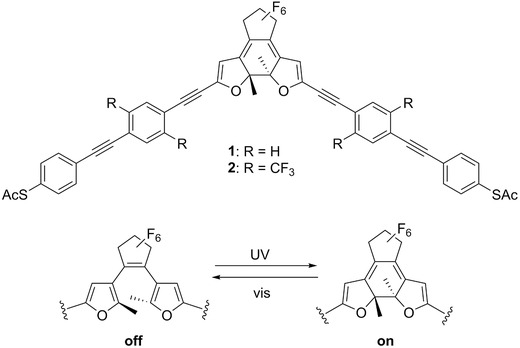
Investigated photochromic switch SMW **1** and **2** with the different side‐groups H and CF_3_. Irradiation with UV (visible) light induces a switching from the off (on)‐state to the on (off)‐state form for both molecules.

We present the electrical characterization of single molecules of oligo(phenylene ethynylene) (OPE)‐embedded difurylperfluorocyclopentenes **1** and **2** at room temperature between gold nanoelectrodes.^27^ These SMWs (**Figure** **1**) consist of a difurylperfluorocyclopentene core equipped with two OPE building blocks, each terminated with a thiol end‐group that forms a reliable molecule‐gold junction.^2^, ^35^ The length of the OPEs (of ≈2 nm) reduces the quenching effect of the excited energy states of the ring‐opened form^9^ due to an efficient decoupling of the diarylethene unit from the gold electrodes and also decreases screening of the light intensity coupled into the connected molecule. This should consequently lead to higher switching efficiencies of a metal–SMW–metal junction. In solution, the quantum efficiency of compound **1** was found to be 0.15 for chloroform and 0.30 for acetonitrile and that of compound **2** was found to be 0.08 for chloroform and 0.29 for acetonitrile, respectively.^22^ In addition, the oxygen‐based furyl core interacts less strongly with the gold contacts than a sulfur‐based thienyl core and thus prohibits the formation of unsuitable configurations of the molecule between the atomic gold contacts.^9^

For clarity, the suffix **off** denotes the ring‐opened “off”‐state and **on** the ring‐closed “on”‐state of the SMWs **1** and **2**. Taking into account measurements with different solvents, the molecules are denoted by a subscripted **t** for toluene and a subscripted **m** for mesitylene.

## 2.2 Data Analysis

Single‐molecule junctions were studied with the technique of MCBJ in liquid environment at room temperature,^27^ which is described in detail in the Experimental Section. For a comprehensive evaluation of the experimental data, two strategies were followed. On the one hand, we recorded conductance traces, so‐called stretching curves, for which the conductance was measured during the bending of the MCBJ under a constant bias voltage (100 mV) and a constant bending speed. This procedure was performed for the molecular species dissolved in toluene and mesitylene, as well as for pure solvents without addition of the molecules (see Figure S4, Supporting Information). The data analysis (described below) then reveals the influence of the molecules on the linear transport properties of the junction. On the other hand, to investigate the conductance in the nonlinear regime and to identify the role of the molecular orbitals contributing to the current path, we gathered current–voltage (*IV*) characteristics in a separate series of measurements again during stretching of the junctions with and without molecules.

Data arising from *IV* characteristics were analyzed using the single‐level transport model, which assumes that one single‐molecular level acts as a transport channel.^25^ CAS‐augmented Hartree–Fock calculations show that the frontier orbitals of the on‐state are energetically well‐separated, nondegenerate levels; thus, they meet the basic conditions for the applicability of the single‐level model.^16^, ^17^, ^18^ Although this assumption seems appropriate in the on‐state, the SMW in the off‐state consists of two π‐systems, which are only weakly coupled in the center. In this state rather tunneling through a series of three barriers (the two metal molecule junctions and the break point of the conjugation in the switching core) should be considered. In addition, the measured currents in those junctions are typically very low making a clear identification of molecular conductance difficult. Thus, within this approach, we focused on single SMWs in the on‐state (assembled as well as in situ closed). The current *I* depending on the voltage *V* is given by the Landauer–Büttiker formalism

(1) $$I(V)=\frac{2e}{h}\int_{-\infty }^{+\infty }T(E,V)\left[f\left(E-\frac{eV}{2}\right)-f\left(E+\frac{eV}{2}\right)\right]dE$$

In this expression, $f(E)=1/[1+\exp((E-\mu )/k_{B}\tau )]$ is the Fermi–Dirac distribution of the electrodes with the chemical potential *μ* and the thermal energy $k_{B}\tau$. The transmission function *T* is given by the Breit–Wigner formula

(2) $$T(E,V)=\frac{4\Gamma_{L}\Gamma_{R}}{{\left[E-E_{0}(V)\right]}^{2}+{\left[\Gamma_{L}+\Gamma_{R}\right]}^{2}}$$

describing the transmission amplitude as a function of the broadening of a single‐molecular level. *E*_0_ defines the position of the energy level of the current leading channel with respect to the Fermi energy. Γ_L/R_ represent the scattering rates, which describe the probability of the electrons to pass the molecule–electrode contact just on the left and just on the right junction, respectively. The sum Γ = Γ_L_ + Γ_R_ equals the level broadening and is related to the electronic interaction strength of the molecule–gold junction. It will be seen that this coupling is rather weak compared with the total binding energy of the molecules on the metallic surface, indicating that contributions to the binding energy are additionally made by orbitals, which do not contribute to the conductance.

For the characterization of the conducting molecular states the level broadening Γ of the conductance channel and the molecular level *E*_0_ were plotted against the transmission *T* in semi‐logarithmic histograms.^2^ All data were normalized to achieve better comparability.

### 2.3 Single SMWs Assembled in the On‐State

**Figure 2:**
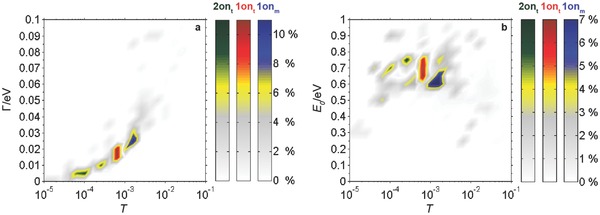
Histograms of a) the level broadening Γ and b) the molecular level *E*_0_ plotted versus transmission *T*. The values were extracted from the single‐level transport model, which was applied to *IV* characteristics of **1on_t_** (1990 curves) and of **2on_t_** (260 curves), dissolved in toluene, and of **1on_m_** (196 curves), dissolved in mesitylene.

For obtaining a precise picture of the electrical behavior of the conductive SMWs and the properties of the formed molecular junctions, we characterized first SMWs that were assembled in the on‐state by applying the single‐level model to *IV* curves. This sets the basis for a detailed understanding of the in situ switching process. **Figure** **2** summarizes the results for the conductive SMWs; the left panel shows the level broadening Γ and the right panel the molecular level *E*_0_ versus transmission *T*.

First, we focus on the influence of the two different side‐groups H (**1on_t_**) and CF_3_ (**2on_t_**). The transmission for **1on_t_** (*T* ≈ 7 × 10^−4^) is higher than for **2on_t_** (*T* ≈ 5 × 10^−5^ to 2 × 10^− 4^). Still, both values are higher than those observed for DAEs with shorter side‐arms and lower switching efficiency.^15^, ^27^ Apparently, decoupling of the excited states of the switching core by the OPE wires does not result in a reduced conductance. Furthermore, the level broadening Γ of **1on_t_** is larger with a peak value of Γ ≈ 20 meV compared with values around Γ ≈ 10 meV for **2on_t_**, which gives a measure for the more conductive molecule–gold junction for **1on_t_**. In contrast to **1**, the CF_3_‐group in **2** extracts electrons from the π‐system due to its high electronegativity, which leads consequently to a lower probability density along the transport channel and reflects a reduced coupling of the molecule to the gold electrodes. Along with the reduced level broadening Γ and the weaker coupling, the CF_3_ groups induce an increase of the energy gap between the current leading channel of the molecule and the Fermi energy. The molecular level *E*_0_ is slightly higher for **2on_t_** with peak values between 0.65 eV and 0.75 eV, compared with 0.6 to 0.75 eV for **1on_t_**. Measurements on **1on_t_** reveal transmission through one well‐defined state, whereas measurements through **2on_t_** indicate that two different states with different *E*_0_ and Γ are found in the conductance. The degree of symmetry of the molecular coupling to the gold electrodes can be given by the symmetry factor *α*, defined by the ratio of the scattering rates Γ_L_ and Γ_R_ (Figure S3, Supporting Information), showing values > 0.7 for the large majority of junctions for all molecular species. This indicates that the binding between metals and molecules is symmetric for the junctions. Therefore, the origin for the two separate conductance states found in case of **2on_t_** is attributed to two different conformations of the molecular junctions. Similar observations of multiple histogram peaks were made for molecular wires, in which the anchoring of the molecules was achieved by thiol–gold bonds.^36^, ^37^ In these publications the different conductance values were attributed to different bonding sites of the sulfur to the Au electrodes, because the molecular wires themselves had no internal degrees of freedom. In our measurements, we can use such differences in conductance values to prove the fact that single molecules, which are contacted by Au nanoelectrodes, are switched in situ, as will be shown below.

We also explored the impact of the solvent on the conductance and the molecular junction. Therefore, we dissolved the closed SMW with the H side‐groups in mesitylene (**1on_m_**). Comparing the data with the results for toluene revealed a higher transmission (*T* ≈ 9 × 10^−3^), an enhanced level broadening (Γ ≈ 20 to 30 meV) and a decreased molecular level energy *E*_0_ with peak values between 0.6 and 0.68 eV. In the following in situ switching experiments we focus on SMWs, which were dissolved in toluene.

### 2.4 In Situ Switching

In the previous section, we gained a detailed view of a single SMW that had been characterized in its on‐state between gold nanoelectrodes. Now, we investigate the in situ switching from the off‐states **1off_t_** and **2off_t_** to the on‐states **1on_t_** and **2on_t_**. Before switching, the solution containing exclusively molecules in the off‐state was brought onto our MCBJ setup and the electrical measurement was started. After this initial characterization, the molecular junction was exposed to UV light (340 nm) for 10 min to initiate the in situ switching of the opened, nonconductive state to the closed, conductive state.

**Figure 3:**
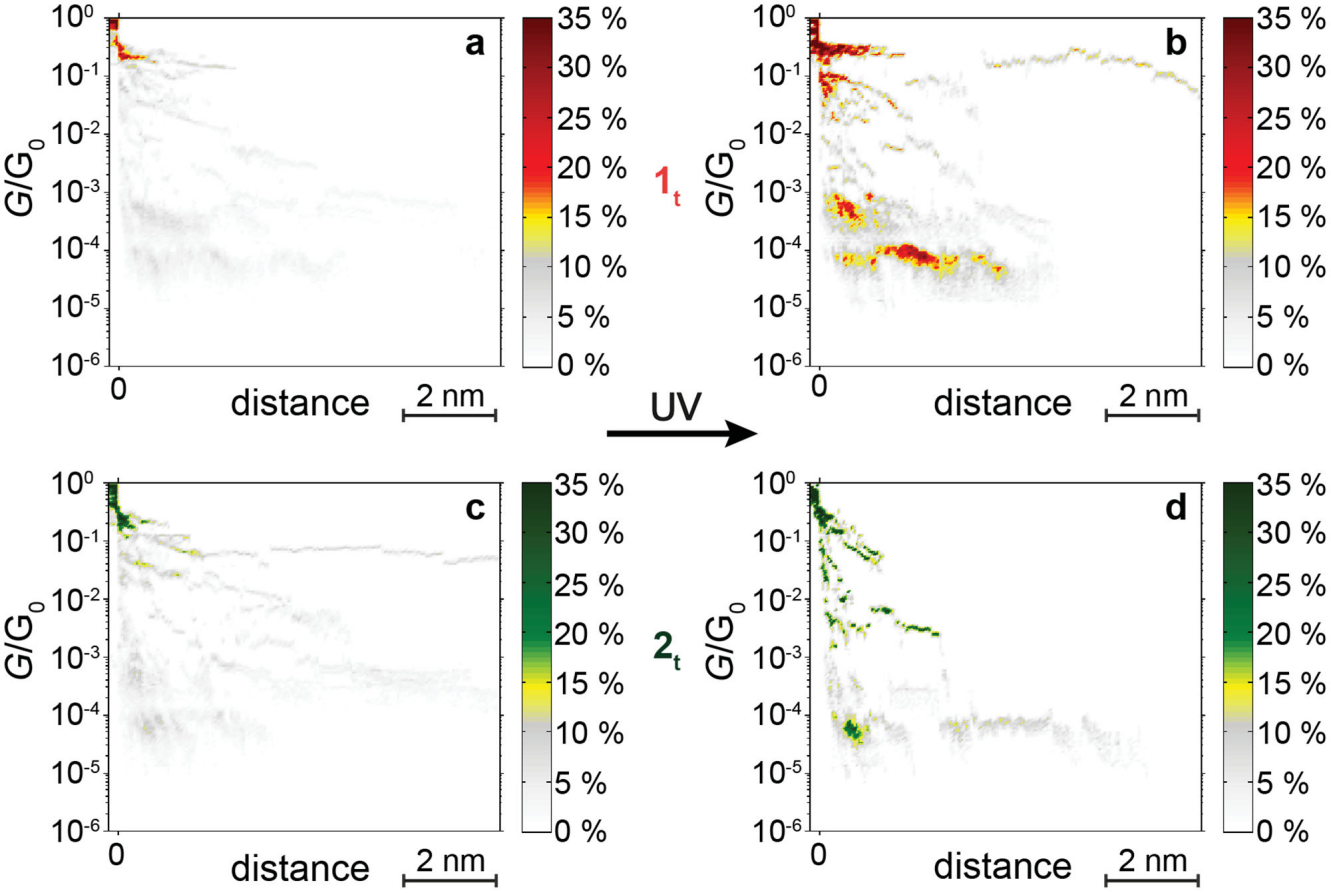
Histograms of a,c) in situ switched **1off_t_** and **2off_t_** to b,d) **1on_t_** and **2on_t_**, obtained from about 50 conductance traces each. After irradiation with UV light, a clear switching to the conductive state can be observed, indicated by defined conductance plateaus with values of *G* ≈ 6 × 10^−5^–1 × 10^−3^ *G*_0_ for **1_t_** and *G* ≈ 2 × 10^−5^–1 × 10^−4^ *G*_0_ for **2_t_**.

This switching of **1off_t_** and **2off_t_** is illustrated in histograms of stretching curves (**Figure** **3**). Before irradiation, both molecular species show similar characteristics. We identify a clear one‐atomic contact at the quantum of conductance *G*_0_ and no defined conductance plateaus of **1off_t_** and **2off_t_** presumably due to a lack of fully conjugated molecular states of **1off_t_** and **2off_t_**. The weak background signal arises mainly from toluene (Figure S4, Supporting Information). After irradiation we observe definite plateaus in the conductance range identified as molecular conductance for the SMWs assembled in the on‐state, which indicates a successful switching to the states **1on_t_** and **2on_t_**. In these plots, the conductance of **1on_t_** lies in the range between 5 × 10^−5^ *G*_0_ and 1 × 10^−3^ *G*_0_, and is higher than for **2on_t_** (*G* ≈ 2 × 10^−5^ to 1 × 10^−4^ *G*_0_) reflecting the electron withdrawing effect of the CF_3_ groups on the delocalized π‐system as discussed above. The findings are also similar to the histograms of conductance traces that are recorded from molecules assembled in the on‐state (Figure S5, Supporting Information), which shows that we observe light‐induced switching of the SMW from the off‐state to the on‐state.

**Figure 4:**
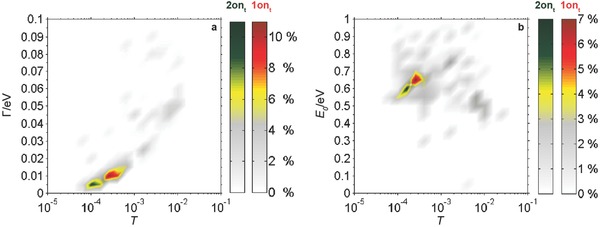
Histograms of a) the level broadening Γ and b) the molecular level *E*_0_ plotted versus transmission *T* after irradiation with UV light. We identify a clear switching from the opened to the closed state due to the defined peaks for **1on_t_** and **2on_t_**. The values are obtained from the single‐level transport model applied to 301 *IV* characteristics for **1on_t_** and 196 for **2on_t_**.

We performed additional analysis of the molecules in the switched on‐state by taking *IV* characteristics and extracting the level broadening Γ and the molecular level *E*_0_ via the single‐level transport model. When we compare these values after in situ switching (**Figure** **4**) with those of molecules assembled in the on‐state (Figure 2), we find agreement in the most significant values. We identify a transmission of *T* ≈ 1 × 10^−4^ to 4 × 10^−4^ for **1on_t_**, which is higher than for **2on_t_** (*T* ≈ 8 × 10^−5^ to 1 × 10^−4^). Furthermore, we obtain values of the level broadening Γ with peak values slightly above 10 meV for **1on_t_** and slightly below 10 meV for **2on_t_** and a value of *E*_0_ ≈ 0.65 eV for **1on_t_**.

There are, however, differences in the results for both components, which can be used to differentiate between switching of the molecules in solution and switching of molecules that are directly contacted by metal electrodes. The molecular level *E*_0_ is lower for the in situ switched **2on_t_** (*E*_0_ ≈ 0.6 eV) than for **2on_t_** (*E*_0_ ≈ 0.65 to 0.75 eV) assembled in the on‐state. The former exhibits only one maximum after in situ switching compared with the two maxima obtained for the SMWs assembled in the on‐state. Furthermore, only one maximum for the values of the level broadening Γ at ≈ 5 meV is found for the in situ switched **2on_t_** while two maxima at 5 and 10 meV are found for the molecules assembled in the on‐state (Figure 2). In addition, the position of the molecular orbital of **1on_t_** when assembled in the on‐state leads to a broad maximum in the histogram, whereas only a narrow distribution arises after the switching process. Evaluation of the asymmetry parameter *α* shows that this spread is caused by a spread of the symmetry of the various junctions with *α* ranging from 0.7 to 1, whereas junctions with **2on_t_** show a spread from 0.9 to 1. Both facts can be understood if we take the mechanical properties of the molecular species into account.

The open bond in the central ring of the opened forms leads to more flexibility along the length of the molecule compared with the ring‐closed form, which is conformationally much stiffer. Therefore, in the ring‐opened form of the SMW the thiol–gold bonds at the connection of the SMW–gold interface have more freedom to find and to move to an equilibrium position. In the ring‐closed form, the mechanical stiffness of the molecules can prevent this process, thus leading to binding in nonequilibrium positions. This can either lead to junctions with lower conductance caused by smaller broadening of the molecular level Γ (**2on_t_**), or to asymmetric junctions because Γ_L_ and Γ_R_ are different due to different binding positions (**1on_t_**). The difference between **1on_t_** and **2on_t_** might be explained by the presence of the side‐groups, which disfavor binding in some positions for **2on_t_** and thus lead to the fact that only two conductance states are found and not a whole range, as it is found in **1on_t_**.

When switching compounds **1** and **2**, the initial position of the thiols at the electrodes will not be changed, since the thiol–gold bond is very robust.^38^ Therefore, we expect mostly symmetric junctions with only one conductance maximum, if the molecules are switched in situ, whereas we expect a wider spread when the molecules are assembled in the on‐state. The experimental finding that only one conductance maximum appears with mostly symmetric junctions (*α* ranging from 0.9 to 1) shows that in situ switching is indeed demonstrated in our experiments. The successful switching of **1off** and **2off** to their closed forms also proves the effective decoupling of the switching core from the gold electrodes.^9^ Regarding the influence of the two different side‐groups, no significant difference in the switching quantum yields was observed.^22^

In contrast to measurements, which have been reported before, switching from the on‐state to the off‐state was not possible in our junctions. Instead, illumination with light, which induces a ring‐opening switching of the molecules in solution,^22^ left the molecule in the on‐state.

### 2.5 Modeling the Switching Process

Spectra obtained from full configuration interaction (CI) calculations for **1** and **2** show that the off‐ and the on‐state do not significantly differ in total energy, but exhibit fundamentally different orbital symmetries.^39^ While the on‐state is characterized by a single HOMO and LUMO level, the off‐states exhibit ideally twofold degenerate frontier orbitals. As suggested from symmetry arguments for the switching core^40^ both forms are stable local minima of the total energy and neither the symmetry change nor the barrier to be overcome during switching may be achieved by simple thermal treatment. The first optically excited states of the off‐ and on‐states also differ significantly in both symmetry and energy. It may, hence, be concluded that ring closure and opening can selectively be triggered optically and follow different mechanistic pathways via a potentially common intermediate as indicated in the supplement.

Regardless of the side‐group, absorption wavelengths of about 550 nm for the ring‐closed switch and of about 340 nm for the ring‐opened forms were calculated for free molecules. These calculations on the free molecules reflect very well the results of experimental optical measurements.^22^ This supports the assumption of an extended π‐system that spans the full length of the ring‐closed on‐state switches, whereas the conjugation is broken in the central moiety of the ring‐opened off‐state switches. In‐line with experimental findings, CF_3_‐substituted side‐chains support a tiny shift of both first excitations to higher wavelengths compared with the unsubstituted molecules. This qualitative description of the molecules already gives sufficient indication that switching occurs optically in both directions, though at different wavelengths.

## 3 Conclusion

In summary, we presented a detailed experimental analysis of single SMWs that were in situ switched from their nonconductive to conductive state. We recorded conductance traces, analyzed the *IV* characteristics in terms of the single‐level model, and identified conductance, level broadening Γ, and molecular level *E*_0_ of single SMW junctions. By comparison of the data with those of SMWs that are assembled in one defined state, evidence for a successful switching process was presented. Moreover, a shift in the energy landscape of the current leading orbital indicates a switching of a molecule that was attached to the Au electrodes before, during, and after the switching process. Exploring the influence on the delocalization of the π‐system, the electron withdrawing CF_3_ side‐groups lead to lower conductance, lower level broadening Γ, and higher molecular level *E*_0_, compared with the H side‐groups.

In total, we were able to contribute a detailed picture of the electrical behavior of single, in situ switched SMWs. The findings confirm that they may act as future building blocks in molecular electronics.

## 4 Experimental Section

For the creation of single‐atom contacts by means of MCBJ, suspended gold nanowires were prepared on a flexible polyimide substrate with dimensions of (100 × 100 × 100) nm^3^ via electron beam lithography, reactive ion beam etching, and reactive ion etching. The molecules were dissolved in toluene or mesitylene with a droplet of aqueous ammonia to remove the acetyl protective groups and were brought onto our sample in a liquid cell. In situ switching of the molecules from the off‐state to the on‐state was accomplished by a UV LED (340 nm), which irradiated the MCBJ with a power of 2 mW cm^−2^ for 10 min during stretching the unbroken nanowire up to approximately 5*G*_0_. In order to allow the light to arrive at the junction, the liquid cell was lifted, whereas droplets of the solution remained on the sample.

All data gathered at fixed bias were summarized in normalized, semi‐logarithmic histograms, where the conductance in units of the quantum of conductance *G*_0_ was plotted against the displacement of the nanoelectrodes without any omission of traces. The distance of the electrodes was determined assuming an exponential decay of the tunneling current between the gold electrodes in solution,^41^ setting the distance to zero at the moment of breaking the contact.^42^ For estimation we assumed the free electron mass and a work function of gold^43^ of 5.1 eV.

## Supporting information

As a service to our authors and readers, this journal provides supporting information supplied by the authors. Such materials are peer reviewed and may be re‐organized for online delivery, but are not copy‐edited or typeset. Technical support issues arising from supporting information (other than missing files) should be addressed to the authors.

Supplementary
