# Supplementary material for: Light‐Induced Switching of Tunable Single‐Molecule Junctions
Source: Adv Sci (Weinh). 2015 Apr 16;2(5):1500017. doi: 10.1002/advs.201500017 (PMC5115361; doi:10.1002/advs.201500017)
Supplement: Supplementary file 1 — Supplementary [file ADVS-2-0j-s001.pdf]

Copyright WILEY-VCH Verlag GmbH & Co. KGaA, 69469 Weinheim, Germany, 2015.

ADVANCED SCIENCE
Open Access

Supporting Information

for *Adv. Sci.*, DOI: 10.1002/advs.201500017

Light-Induced Switching of Tunable Single-Molecule Junctions

*Torsten Sendler, Katharina Luka-Guth, Matthias Wieser, Lokamani, Jannic Wolf, Manfred Helm, Sibylle Gemming, Jochen Kerbusch, Elke Scheer, Thomas Huhn, and Artur Erbe**

Copyright WILEY-VCH Verlag GmbH & Co. KGaA, 69469 Weinheim, Germany, 2013.

Supporting Information

Light-Induced Switching of Tunable Single Molecule Junctions

*Torsten Sendler, Katharina Luka-Guth, Matthias Wieser, Lokamani, Jannic Wolf, Manfred Helm, Sibylle Gemming, Jochen Kerbusch, Elke Scheer, Thomas Huhn, and Artur Erbe**

Supporting information - table of contents

1. Data analysis – normalization of histograms

2. *IV*-characteristics

3. Symmetry factor

4. Conductance histograms

4.1 Solvents

4.2 SMWs assembled in the ring-closed state

5. Computational details

6. References

1. Data analysis

Conductance traces were recorded under a constant bias voltage of 0.1 V. Thereby the opening speed of the break junction was chosen very low to extract a high number of data points from each single stretching curve. In general the lifting movement of the seal within the conductance range below 30 G_0 was 0.6 µm/min at a displacement ratio of $5.9 \cdot 10^{-6}$.[1] Afterwards the data was plotted in normalized histograms to the bin with the highest amount of data points to allow for an exact comparison of the presented results. For the extraction of the level broadening Γ and the molecular level E_0, *IV*-characteristics were fitted by applying the single level transport model.[2,3] The data were plotted in semi-logarithmic, color-coded histograms and normalized to the total amount of data points of each histogram (**Figure S1**).

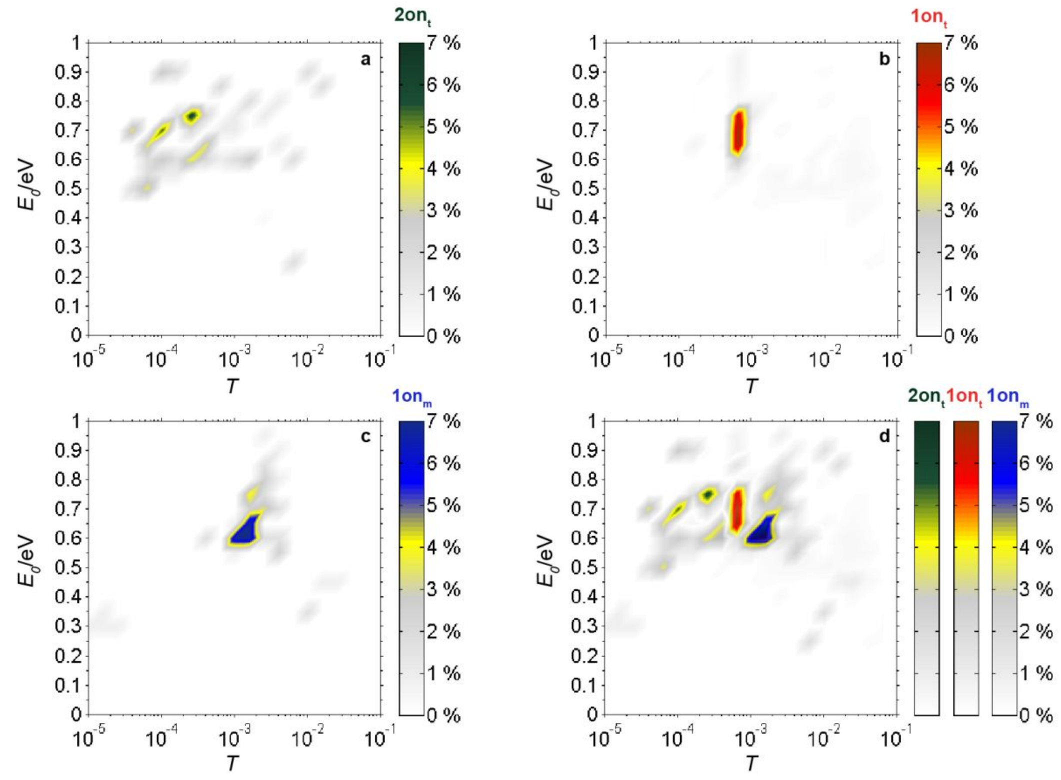

Figure S1. a) – c): normalized histograms of the molecular level E_0 for single, assembled, closed SMWs with -H and -CF_3 side-groups that were dissolved in toluene (**1on$_t$**, **2on$_t$**) as well as mesitylene (**1on$_m$**). (d): results summarized in one plot. The values were obtained from the single level transport model applied to 1990 (**1on$_t$**), 260 (**2on$_t$**) and 196 (**1on$_m$**) *IV*-characteristics.

2. *IV*-characteristics

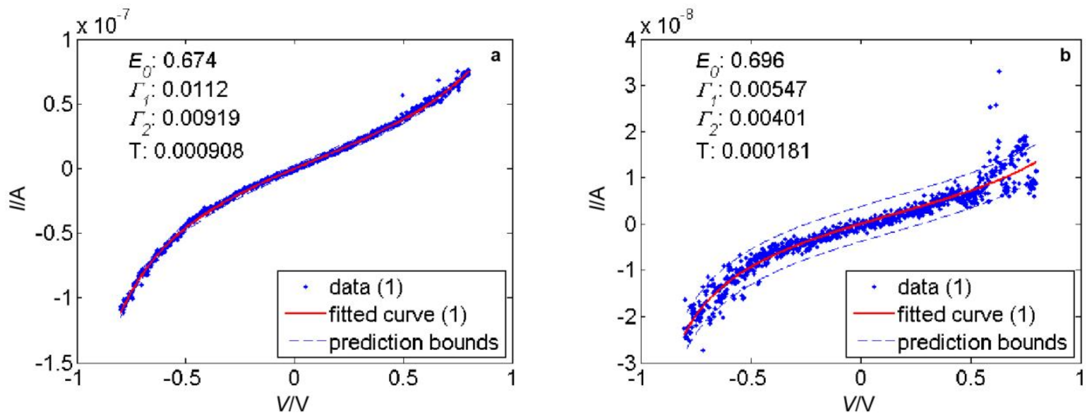

Figure S2. *IV*-curves of single SMW junction with -H (a) and -CF_3 (b) side-groups. The blue points correspond to experimental data, the red curve to the fit from the single level model. The values for Γ, E_0 and T illustrate the expected behavior for the different side-groups in an excellent way. The slight asymmetry in the *IV*-characteristic of the SMWs with the CF3 side-groups (right panel) indicates the development of two peaks in Figure 2 in the main paper.

3. Symmetry factor α

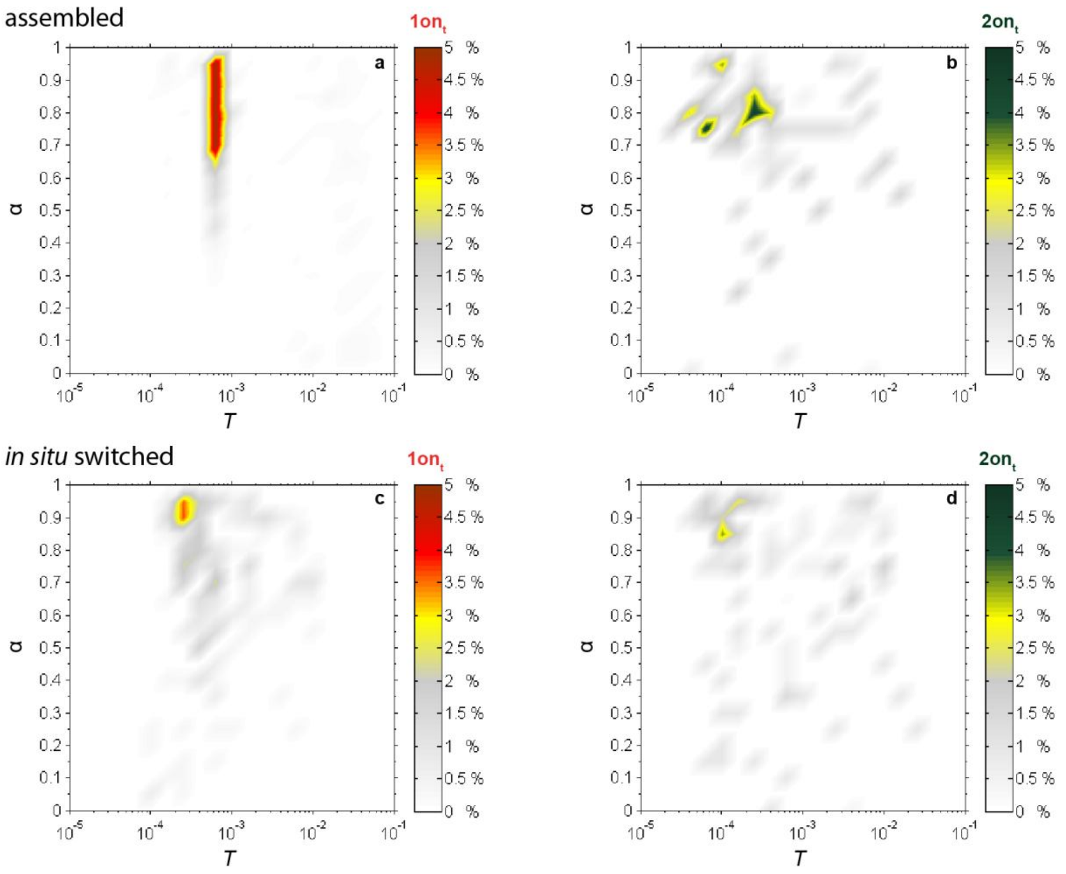

Figure S3. Normalized histograms of the symmetry factor α for assembled (a, b) and *in situ* switched (c, d) SMWs with -H (**1on$_t$**) and -CF_3 (**2on$_t$**) side-groups, given by the ratio of the scattering rates Γ_L and Γ_R. We divided the smaller of the two values by the bigger such that α adopts values between 0 and 1. The latter indicates is a highly symmetric molecule gold junction. The values of the assembled SMWs were extracted from 1990 (**1on$_t$**) and 260 (**2on$_t$**), the values of the *in situ* switched SMWs from 301 (**1on$_t$**) and 196 (**2on$_t$**) *IV*-curves.

4. Conductance histograms

4.1 Solvents

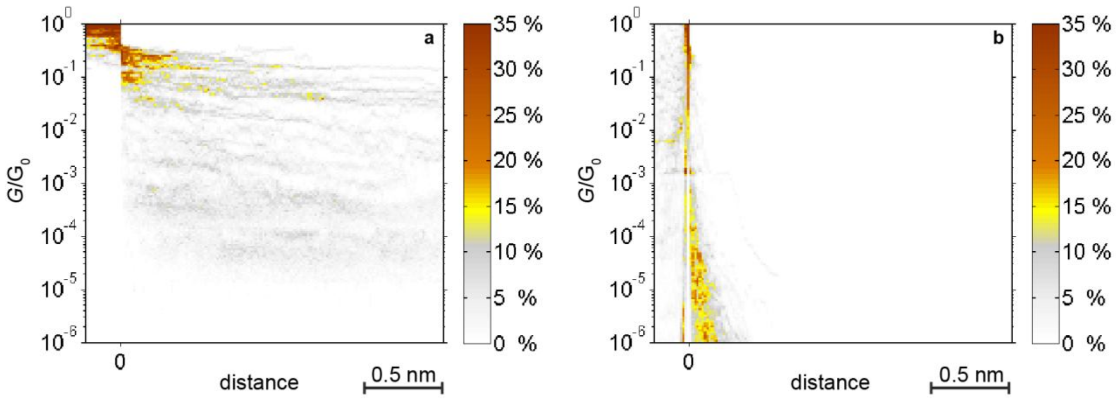

Figure S4. Normalized histograms of conductance traces of toluene (a, 87 traces) and mesitylene (b, 570 traces) without dissolved molecules.

4.2 SMWs assembled in the closed state

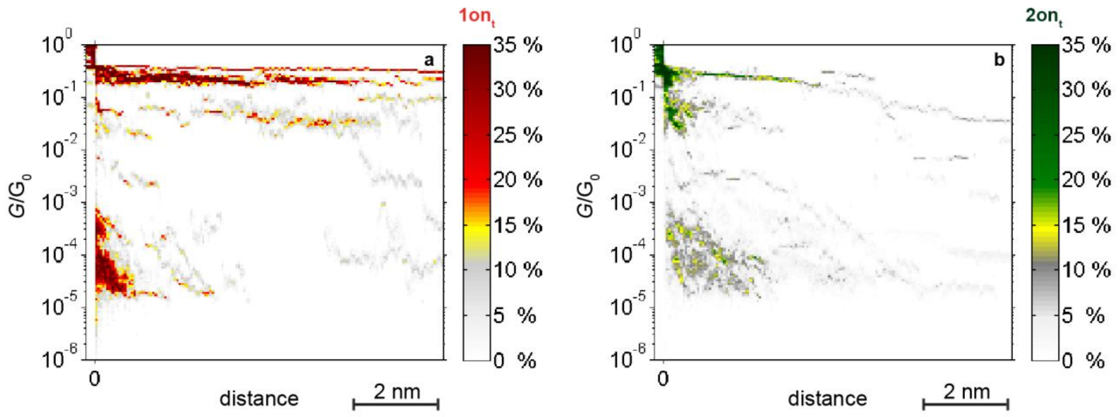

Figure S5. Normalized histograms of conductance traces taken from SMWs with -H (a, 296 traces) and -CF_3 (b, 117 traces) side-groups that were assembled in the conductive state. The two pronounced plateaus between $G \approx 10^{-3}$ - 10^{-5} G_0 for the -H side-groups may arise from the thiol linkers that create two ways of molecule gold junctions. This effect may be suppressed for the SMW with -CF_3 side-groups due to their lower conductance and a different conformation.

5. Computational details

For the quantum chemical investigations of the present rather large molecules the semi-empirical program package MOPAC in the PM7 parameterization was chosen.[4] The approach was evaluated by comparison with literature values from full ab-initio CAS(10,10) and CAS(12,12) calculations.[5] The energies and states involved in the ring opening and closure mechanism of the central difurylcyclopentene moiety agree within 0.1 eV, which is sufficiently accurate for the present qualitative study.

In order to calculate the excitation frequencies of the ring-opened off-state and the ring-closed on-state the geometries of **1on**, **1off**, **2on**, and **2off** were optimized at the multi-reference level, including all 2p-based valence orbitals in the configuration interaction ansatz. All four molecules are stable ground state structures of about 32 Å distance between the two S atoms; the ring-opened forms are more stable than the ring-closed ones by 0.12 eV (**1**) and 0.29 eV (**2**). A rotation of the side-groups around the bond between the furyl group and the central cyclopentene unit is energetically unfavorable for all structures by on average 0.1 eV per rotated bond. All four molecules exhibit a singlet ground state with single HOMO and LUMO levels, which span the whole molecule in the case of the ring-closed forms. In contrast, for the ring-opened forms doubly degenerate HOMO and LUMO levels are obtained, which are each confined to only one side of the SMW. In very good qualitative agreement with the experiment these results indicate that only the ring-closed forms **1on** and **2on** exhibit molecular states suitable for ballistic transport, whereas the aromatic system is broken in two parts for the ring-opened switches.

The single-reference HOMO-LUMO gaps of the ring-opened forms are larger than the ones of the ring-closed forms by 0.7 eV (**1**) and 0.5 eV (**2**), and the gaps of the substituted compounds **2on** and **2off** are smaller than the ones of **1on** and **1off** by 0.3 eV and 0.5 eV, respectively. These findings indicate that the substitution of the phenyl rings with -CF_3 substituents changes neither the localization pattern nor the general orbital splitting. Due to their electron-

withdrawing character the -CF_3 groups, however, modify the aromatic nature of the frontier orbitals: the reduced HOMO-LUMO gaps of compounds **2** indicate that the alignment with the Fermi level of the lead and also the coupling strength of compounds **1** and **2** will differ. The first singlet excitation energies obtained from full CI calculations are in very good agreement with experimental observations for the molecules in toluene solution.[6] Visible light can excite the ring-closed forms at about 530 nm (**1on**) and 550 nm (**2on**), whereas UV light of about 400 nm and 420 nm is required to excite the ring-opened forms **1off** and **2off**. This reflects the delocalization of the frontier orbitals of the ring-closed switches **1on** and **2on** and the confinement of the aromatic system to subunits of the ring-opened molecules **1off** and **2off**.

Figure S6 shows the energies of the ground state and the first two excited singlet states as a function of the C-C atom distance at the central part of the switch, which is chosen here as reaction coordinate. Apart from this reaction coordinate all degrees of freedom were fully optimized at the CI level. The transition between ring-closed and ring-opened states occurs in a conrotatory manner[7] and involves an energy barrier of 2.2 eV for the unsubstituted molecule 1, which agrees well with the barrier of 2.17 eV calculated by Boggio-Pasqua et al. for the central switching unit with the CAS-SCF method (4). A slightly higher value of 3.0 eV for **2** confirms the stability of both molecules against thermally induced ring closure and opening isomerization. The transition occurs at values of the reaction coordinate of about 2.20 Å (**1**) and 2.30 Å (**2**), where the canting of the central unit with respect to the two arms sets in. There, the optimum sulfur-to-sulfur distance shrinks by about 2 Å with respect to both reactant and product. Along with the arguments given in (4) for a reduced quantum yield of the ring opening reaction due to the presence of an energy barrier and several conical intersections on the first excited energy surface this geometry change may be another reason for the low propensity of the molecule to switch back to the opened form *in situ*.

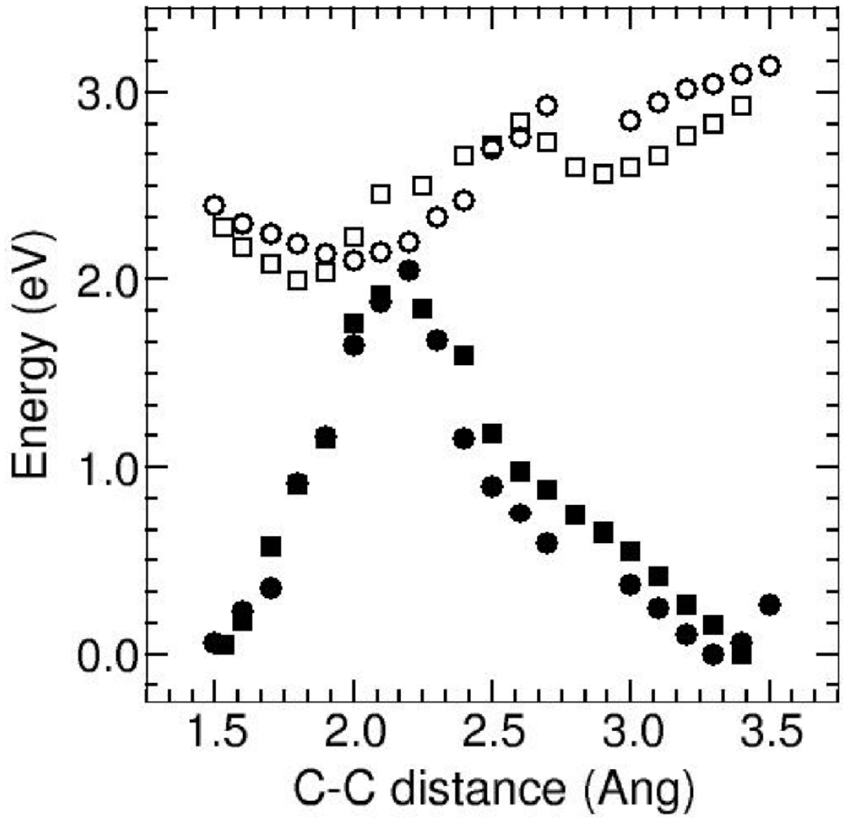

Figure S6. Energies of the ground state (filled symbols) and the first excited singlet states (open symbols) of molecules **1** (squares) and **2** (circles) with respect to the lowest ground state energy. The energies are calculated by a full CI approach within a semi-empirical Hartree-Fock-type treatment.[4]

6. References

[1] J. M. van Ruitenbeek, A. Alvarez, I. Piñeyro, C. Grahmann, P. Joyez, M. H. Devoret, D. Esteve, C. Urbina, *Rev. Sci. Instrum.* **1996**, *67*, 108.

[2] Y. Kim, T. Pietsch, A. Erbe, W. Belzig, E. Scheer, *Nano Lett.* **2011**, *11*, 3734.

[3] L. A. Zotti, T. Kirchner, J. C. Cuevas, F. Pauly, T. Huhn, E. Scheer, A. Erbe, *Small* **2010**, *6*, 1529.

[4] MOPAC2012, J. J. P. Stewart, "Computational Chemistry," can be found under HTTP://openmopac.net, **2012**.

[5] M. Boggio-Pasqua, M. Ravaglia, M. J. Bearpark, M. Garavelli, M. A. Robb, *J. Phys. Chem. A* **2003**, *107*, 11139.

[6] J. Wolf, I. Eberspächer, U. Groth, T. Huhn, *J. Org. Chem.* **2013**, *78*, 8366.

[7] S. Nakamura, M. Irie, *J. Org. Chem.* **1988**, *53*, 6136.
